# Influence of RF Sputtering Pressure and Power on the Microstructure of Sb Thin Films

**DOI:** 10.3390/ma19143119

**Published:** 2026-07-21

**Authors:** Sheyda Uc-Canche, Eduardo Camacho-Espinosa, Mariely Loeza-Poot, Ricardo Mis-Fernández, Eduardo Flores

**Affiliations:** 1 Departamento de Física Aplicada, Centro de Investigación y de Estudios Avanzados CINVESTAV-Unidad Mérida, Mérida 97310, Mexico; mariely_lop@hotmail.com (M.L.-P.); rimis@cinvestav.mx (R.M.-F.); 2 División Industrial, Universidad Tecnológica Metropolitana, Mérida 97279, Mexico; 3 Departamento de Energías Renovables, Universidad Abierta y a Distancia de México, México City 03330, Mexico; ed_cam_es@hotmail.com

**Keywords:** Sb thin films, RF-Sputtering power, microstructure, working pressure

## Abstract

**Highlights:**

- A critical RF power threshold (60 W) triggers the amorphous-to-rhombohedral transition in Sb thin films.
- RF-Sputtering conditions enable texture engineering, driving orientation redistribution from (003) to (104).
- Enhanced adatom mobility promotes grain coarsening from ~40 to ~110 nm, reshaping film morphology.
- Microstructural optimization reduces resistivity by several orders of magnitude to the 10^−4^ Ω·cm range.
- XPS reveals a chemically stable Sb thin film, demonstrating that transport evolution is structurally driven.

**Abstract:**

Antimony (Sb) thin films are critical precursors for next-generation chalcogenide-based optoelectronics. However, the synergistic effects of sputtering power and pressure on their growth dynamics are not yet fully understood. The objective of this study is to establish the conditions for controlling the growth process of Sb films. To this end, we systematically investigated the interplay between RF-Sputtering parameters and film functionality. Samples were deposited as ~800 nm-thick films using RF-Sputtering, varying the power (50–70 W) and working pressure (10–25 mTorr), followed by comprehensive structural (XRD), morphological (SEM), chemical (XPS), and electrical characterization. A critical power threshold of 60 W was identified for the amorphous-to-crystalline transition, at which the films adopt a rhombohedral phase. Increasing the power to 70 W triggered a significant texture redistribution toward the (104) plane and an increase in grain size from 40 nm to 110 nm. Consequently, electrical resistivity dropped by several orders of magnitude, reaching a minimum of ~10^−4^ Ω cm due to enhanced grain connectivity and reduced boundary scattering and the transition from semiconductor-type conductivity to metallic-type conductivity. XPS analysis confirmed that these variations are driven by microstructural evolution rather than chemical changes, as the films maintain a stable metallic character beneath a nanometric surface oxide. These findings establish a direct correlation between plasma conditions and material properties. This strategic framework supports the optimization of Sb-based precursor layers in high-efficiency thin-film technologies.

## 1. Introduction

Antimony (Sb) is a fascinating semimetallic element that crystallizes in a rhombohedral structure (space group R3m) [1]. This greyish, lustrous metalloid exhibits physical properties that are highly sensitive to its microstructural characteristics. In thin-film form, parameters such as grain size, crystallinity, preferred orientation (texture), and defect density play a decisive role in determining the material’s optoelectronic behavior. Given that Sb has a relatively low melting point (630 °C) and a versatile valence state that allows for various oxidation states [1,2], precise control of growth conditions is essential for tailoring its properties toward specific technological applications.

Sb has historically been valued for its ability to harden metal alloys and its fundamental role in the flame-retardant industry, as well as in the fabrication of infrared detector diodes and high-speed, low-power electronic devices [3,4]. However, its current relevance has shifted toward the advanced semiconductor sector, as it serves as the foundation for understanding and fabricating high-impact derivative materials, such as antimony chalcogenides (Sb_2_S_3_ and Sb_2_Se_3_) [5,6,7]. These compounds are emerging as highly efficient light-absorbing materials due to their tunable direct and indirect energy band gaps (between 1.0 and 1.8 eV) and high absorption coefficients, making them ideal for next-generation photovoltaic devices and optical sensors [8,9,10].

Even though elemental antimony has been investigated for decades and many of its fundamental properties are considered well-established, recent literature has focused predominantly on its compounds, often overlooking Sb as a standalone material [8,11]. Nevertheless, historical experimental limitations (which often resulted in poor control overgrowth kinetics) can now be overcome thanks to advances in deposition and characterization technologies. Modern structural and chemical analysis tools now provide unprecedented resolutions to examine microstructural features that were previously inaccessible. Consequently, there is a clear need to revisit elemental Sb thin films under updated experimental approaches, not only to validate reported behaviors but also to uncover new growth mechanisms and structure–property relationships.

Within fabrication techniques, Radio Frequency (RF) magnetron sputtering stands out for offering rigorous control over plasma conditions and energy transfer to the growing film. In semimetallic films obtained by this technique, the sputtering power and working pressure are the critical variables governing adatom mobility and nucleation density [9,10]. Although previous studies on related materials suggest that increasing power favors crystallinity while pressure modulates the mean free path of sputtered species [12,13,14,15,16,17,18], the complex interaction between both parameters (specifically for Sb) has not yet been systematically studied. Furthermore, Sb exhibits a phase transition from an amorphous to a crystalline state upon reaching a critical thickness and temperature [19,20,21]. However, this transition has not been sufficiently investigated under specific sputtering conditions.

Previous studies on RF-sputtered Sb thin films have primarily focused on isolated aspects of film growth, such as crystallization behavior, deposition rate, or the influence of a single deposition parameter. Moreover, most of these studies were conducted several decades ago using characterization techniques with considerably lower spatial and chemical resolution than those currently available. To the best of our knowledge, no systematic investigation has correlated the combined influence of RF power and working pressure with the structural, morphological, chemical, and electrical evolution of metallic Sb thin films. Furthermore, the progressive morphological evolution associated with these deposition parameters has not been comprehensively documented. In view of this, a scientific gap exists regarding how the interplay between moderate working pressures and variation in sputtering power affects the structural, morphological, and electrical evolution of Sb, particularly in contexts where these films are employed as precursor layers in complex device architectures. In such applications, subtle changes in Sb microstructure can strongly influence subsequent processing steps and device performance, underscoring the need for a systematic understanding of these synergistic effects. Therefore, in the present work, Sb thin films were systematically deposited by RF-Sputtering under controlled variations of deposition power and working pressure. The effects of these parameters on the structural, morphological, chemical, and electrical properties were comprehensively investigated. By systematically correlating RF power and working pressure with the structural, morphological, chemical, and electrical evolution of Sb thin films, this work provides one of the first comprehensive descriptions of the coupled growth mechanisms governing RF-sputtered Sb films.

## 2. Materials and Methods

Antimony (Sb) thin films were deposited onto Corning 2947 glass substrates using the radiofrequency (RF) magnetron sputtering technique. A 4N-purity antimony target (K.J. Lesker) was employed as the sputtering source. Prior to deposition, the glass substrates were ultrasonically cleaned in a sequential bath of detergent, methanol, and acetone, followed by drying with compressed air.

The vacuum chamber was initially evacuated to a base pressure of approximately 1 × 10^−5^ Torr. Prior to each deposition, the Sb target was pre-sputtered for 5 min using a power ramp of 10 W every 20 s up to 50, 60, or 70 W to remove surface contaminants and stabilize plasma conditions. The deposition parameters (specifically, argon working pressure and RF discharge power) were systematically varied. The selected pressure (10–25 mTorr) and RF power (50–70 W) ranges were established from preliminary deposition trials within the stable operating window of the sputtering system, where reproducible film growth and significant microstructural variations were observed. Preliminary XRD measurements revealed the onset of the amorphous to crystalline transition within this RF power range (See Supplementary Materials, Figure S1). The first set of films was deposited at pressures of 10, 20, and 25 mTorr, all with a fixed RF power of 50 W. Subsequent deposition series were carried out at RF powers of 60 W and 70 W, each using the same set of working pressures (10, 20, and 25 mTorr). To establish the deposition rate (20–32 nm/min) under each sputtering condition, reference films were initially deposited at room temperature for 20 to 45 min. Based on these measurements, the deposition time was adjusted for each condition to obtain Sb films with a thickness of approximately 800 nm. The thickness was intentionally selected to ensure that all samples were compared within the same thickness regime, allowing the observed variations in crystallinity, texture, morphology, and electrical resistivity to be attributed exclusively to the RF sputtering power and working pressure [20]. At lower thickness, Sb films are known to exhibit thickness-dependent effects, including incomplete grain and amorphous-to-crystalline transition behavior, which could mask the influence of the deposition conditions investigated in this work. Moreover, a thickness of 800 nm provides continuous films with sufficient material volume for reliable XRD, SEM, XPS, and four-point probe measurements, while avoiding unnecessarily long deposition times. Therefore, this thickness represents a practical compromise between minimizing thickness-related effects and ensuring robust structural, morphological, chemical, and electrical characterization. The complete set of deposition parameters is summarized in Table 1, enabling a systematic evaluation of the influence of power and pressure on film growth.

The diffraction patterns were obtained using X-ray diffraction (XRD) on a Siemens D-5000 diffractometer (Munich, Germany); the measurement was performed in 2θ sweeping from 10 to 80 degrees with a scan speed of 0.02 degree/3 s, using a Cu kα tube (λ = 1.5406 Å). Additionally, the crystallite size and lattice parameters were extracted from the XRD patterns using standard analytical methods. The morphological properties of the films were analyzed using field emission scanning electron microscopy (FE-SEM, JSM-7600F, JEOL Ltd., Tokyo, Japan) (JEOL JSM-7600F). The average grain size was determined from the SEM images using the open-source software ImageJ (National Institutes of Health, Bethesda, MD, USA, https://imagej.net/ij/ (accessed on 16 July 2026)). Prior to the analysis, the image scale was calibrated using the scale bar provided in each SEM image. Grain size measurements were then performed manually by drawing a straight line across the maximum apparent diameter of individual grains, and the corresponding lengths were obtained using the measurement tools available in ImageJ. For each sample, multiple grains distributed over different regions of the micrograph were measured, and the average value was reported as the representative grain size. The sheet resistance of the films (Ω/□) was measured using the four-point probe method with a Keithley 2420 Source-Meter and a custom-built four-point probe setup. The electrical resistivity was subsequently calculated from the measured sheet resistance and film thickness. The film thickness was measured using a Veeco DEKTAK 150 surface profilometer (Veeco Instruments Inc., Plainview, NY, USA). A stylus scan length of 1 mm was employed to determine the step height, providing a representative measurement of film thickness and confirming surface uniformity. The chemical composition was investigated through X-ray photoelectron spectroscopy (XPS) using a Thermo Scientific K-Alpha system (Thermo Fisher Scientific, East Grinstead, West Sussex, UK) equipped with an Al Kα X-ray source.

## 3. Results

### 3.1. Structural Analysis

**Figure 1:**
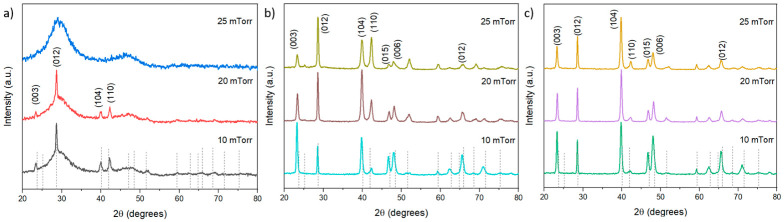
XRD patterns of Sb thin films deposited by RF-Sputtering under different deposition conditions: (**a**) 50 W, (**b**) 60 W, and (**c**) 70 W, for working pressures of 10, 20, and 25 mTorr.

The structural evolution of the Sb thin films was investigated by X-ray diffraction (GI-XRD). Figure 1 displays the diffractograms for films deposited at (a) 50 W, (b) 60 W, and (c) 70 W under different working pressures (10, 20, and 25 mTorr).

A clear dependence of the structural characteristics on both RF power and working pressure is observed. At 50 W (Figure 1a), the patterns are dominated by broad, low-intensity features, indicative of low crystallinity. However, a pressure-dependent evolution is evident: at 25 mTorr, the pattern shows a diffuse halo, while decreasing the pressure to 20 and 10 mTorr leads to the progressive emergence of a broad reflection around the (012) plane, together with weak contributions from the (003) and (104) reflections. This behavior suggests the development of short-range order and incipient nanocrystallinity rather than a fully amorphous structure.

**Figure 2:**
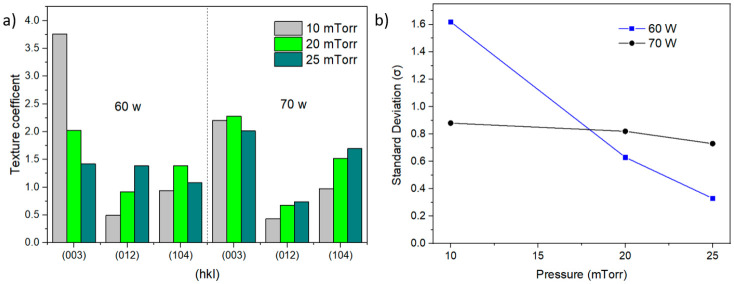
(**a**) TC and (**b**) σ calculated for the samples deposited at different powers and pressures.

Increasing the RF power from 60 W to 70 W (Figure 1b,c) promotes the formation of a polycrystalline rhombohedral Sb phase. All diffraction peaks can be indexed to metallic rhombohedral antimony (PDF No. 00-085-1322, space group R–3m), with the main reflections located at approximately 23.7°, 28.7°, 40.1°, 41.9°, 47.1°, and 48.4°, corresponding to the (003), (012), (104), (110), (015), and (006) crystallographic planes, respectively. No additional diffraction peaks associated with secondary phases or antimony oxides were detected within the resolution of the XRD measurements. To quantify these orientation shifts, the Texture Coefficient (TC) and its standard deviation (σ) were calculated using Equations (1) and (2) [22,23]:

(1) $$TC=\frac{I_{\left(hkl\right)}/I_{0(hkl)}}{\left(1/N\right)\left[\sum_{N}I_{\left(hkl\right)}/I_{0(hkl)}\right]}$$

(2) $$\sigma =\sqrt{\sum \frac{1}{n}{\left(TC-1\right)}^{2}}$$

where *I*_0(*hkl*)_ and *I*_(*hkl*)_ correspond to the standard and experimental peak intensities, respectively, and N is the number of reflections considered. In this context, *TC* = 1 represents a random distribution, while higher values indicate a stronger degree of texture. The results, summarized in Figure 2a,b, confirm that at 60 W and 10 mTorr, the films possess a strong preferential orientation along the (003) plane (*TC* = 3.76 and σ = 1.62).

As pressure increases or power shifts to 70 W, σ generally decreases, reflecting a transition toward a more randomized or redistributed crystallographic texture, specifically favoring the (104) orientation at higher energies. The decrease in σ reflects a transition toward a more isotropic texture, driven by increased scattering at higher pressures and enhanced surface diffusion at higher RF power, both of which redistribute the preferred crystallographic orientations in accordance with established sputtered thin film growth models [24].

Beyond grain orientation, it is essential to evaluate the stability of the unit cell and the physical dimensions of the crystallites. The lattice parameters (a and c) and the crystallite size (D) were determined using the hexagonal representation of the rhombohedral [25,26,27] system and the Debye–Scherrer equation [23,24,25,26,27,28], Equations (3) and (4), respectively:

(3) $$D=\frac{k\lambda }{\beta_{hkl\;\;}\cos\theta }$$

(4) $$d_{\left(hkl\right)}={\left[\left(4/3a^{2}\right)\left(h^{2}+k^{2}+hk\right)+\left(l^{2}/c^{2}\right)\right]}^{-1/2}$$

where *D* is the average crystalline size, *k* is the factor shape of the crystal (0.9), *λ* is the X-Ray wavelength (0.15406 nm), *β_hkl_* is the full width at half maximum (FWHM) of the diffraction peak, and *θ* is the Bragg angle. The indices *h*, *k*, and *l* correspond to the Miller indices of the crystal planes, while a and c are the lattice parameters of the hexagonal representation, and *d*_(*hkl*)_ is the interplanar spacing.

**Figure 3:**
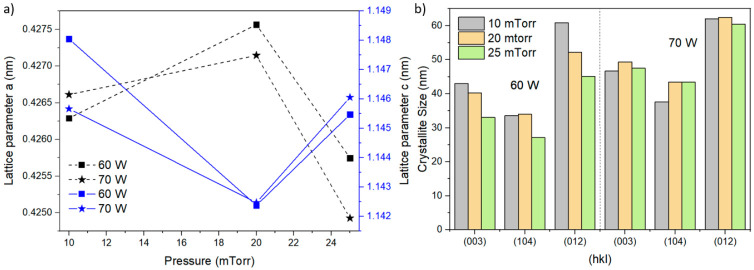
(**a**) Lattice parameter and (**b**) crystallite size of the sample at different power grown and work pressure.

Figure 3a shows that, compared with the standard values (a = 0.4307 nm, c = 1.1273 nm), the experimental lattice parameters exhibit a slight decrease in a and an increase in c, indicating an anisotropic distortion in the hexagonal representation of rhombohedral Sb. Despite these variations, the unit cell volume exhibits only a minor decrease (~1%), suggesting that the overall crystal structure is largely preserved and that the observed changes are primarily associated with anisotropic lattice distortion rather than significant volumetric contraction. The observed contraction along the a-axis and expansion along the c-axis may reflect preferential incorporation of residual stress during film growth, consistent with the compressive in-plane stresses commonly reported in sputtered thin films at moderate working pressures. The near-constant unit cell volume confirms that these distortions represent an elastic response of the lattice rather than a change in phase or stoichiometry.

In contrast, the crystallite size (Figure 3b) is strongly influenced by the plasma conditions. In general, D increases with RF power, reaching its maximum at 70 W, which can be attributed to enhanced adatom mobility. Notably, the (012) plane consistently exhibits the largest crystallite size across all conditions, even when it is not the dominant orientation. This behavior indicates a decoupling between texture and grain growth: while RF power and working pressure control the preferential orientation of the films, the extent of crystallite development is primarily governed by the energy available for atomic diffusion and grain boundary migration.

Overall, the XRD analysis demonstrates that RF power and working pressure play distinct but complementary roles in determining the structural properties of Sb thin films. These results establish clear correlations between processing conditions and structural evolution, providing fundamental insight into the growth mechanisms of RF-Sputtered Sb thin films and supporting the identification of suitable processing windows for their integration into advanced thin-film technologies.

### 3.2. Morphological Properties

**Figure 4:**
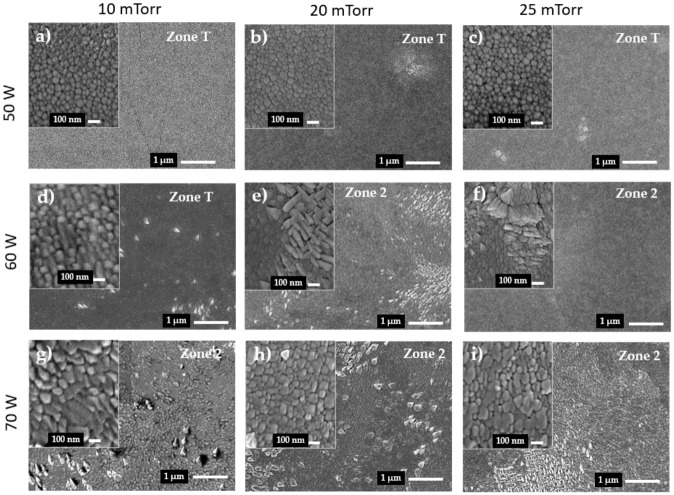
Surface FE-SEM images of the samples deposited at different pressures and sputtering-power: 50 W (**a**–**c**), 60 W (**d**–**f**), and 70 W (**g**–**i**).

The surface morphology of the Sb thin films as a function of RF-Sputtering power and working pressure is summarized in the 3 × 3 matrix shown in Figure 4, where rows correspond to increasing RF power and columns to increasing working pressure. High-magnification inset (100,000×) highlights grain boundaries and local geometry. While both parameters influence film growth, variations along the sputtering power axis produce more pronounced morphological changes than those associated with pressure, indicating that sputtering power is the dominant factor governing grain evolution. This high sensitivity to RF power can be attributed to the intrinsic thermophysical properties of Sb. Due to its relatively low melting point (630 °C) and high vapor pressure under vacuum, Sb exhibits enhanced atomic mobility even at moderate temperatures [29]. Consequently, small increments in RF power (through increased ion bombardment and localized heating) are sufficient to markedly alter surface diffusion and grain growth kinetics.

Along the main diagonal of the matrix (Figure 4a,e,g), a clear morphological progression is observed, evolving from quasi-spherical grains to faceted acicular structures and finally to polygonal, Voronoi-like grains. This evolution is consistent with the Thornton structural zone model, where film morphology is governed by the balance between adatom mobility and deposition conditions.

At low power (50 W; Figure 4a–c), the films consist of densely packed quasi-spherical grains with an average size of ~40 nm, characteristic of Zone 1/Zone T, where limited surface diffusion leads to compact, fine-grained, or poorly crystallized structures [30]. At this power, the three samples exhibit similar morphologies, although the film deposited at 20 mTorr appears slightly more compact. This quasi-spherical morphology is consistent with the amorphous or quasi-amorphous structure observed by XRD, indicating restricted adatom mobility and non-directional growth.

At intermediate power (60 W; Figure 4d–f), the films enter a transition regime between Zone T and Zone II, where increased adatom mobility promotes grain growth and faceting. The morphology evolves toward larger (~100 nm), faceted grains, with a pronounced acicular structure particularly evident at 20 mTorr, reflecting anisotropic growth along preferred crystallographic directions in rhombohedral Sb. In this regime, working pressure enhances thermalization of sputtered species, favoring elongated features.

The observed morphological evolution is consistent with the structural changes identified by XRD and reflects the combined influence of RF power and working pressure. At low power, the films exhibit small, isotropic grains associated with low crystallinity, whereas increasing power leads to more anisotropic grains, indicating the development of preferential growth directions and improved crystalline order. At the highest power, the formation of larger, well-defined grains suggests enhanced atomic diffusion and increased crystallinity. Although pressure plays a secondary role, it modulates surface diffusion and grain arrangement, thereby supporting the trends observed in XRD.

### 3.3. Electrical Analysis

**Figure 5:**
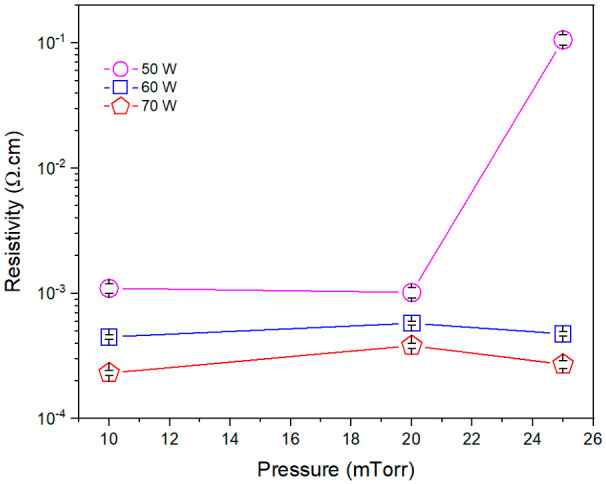
Resistivity calculated for Sb samples deposited at different sputtering powers and working pressures.

The electrical resistivity (*ρ*) of the Sb thin films as a function of working pressure and RF power is presented in Figure 5. The numerical values of resistivity and their corresponding standard deviations are provided in Table S1 of the Supplementary Material. In this work, the sheet resistance of the sputtered Sb thin films was measured using the four-point probe method, and the resistivity was determined according to [31,32]:

(5) $$\rho =\;f_{1}\cdot \frac{\pi }{\ln2}\cdot t\cdot R$$

where *ρ* is the electrical resistivity, *t* is the film thickness, *R* is the sheet resistance, and *f*_1_ is a geometrical correction factor that depends on the probe spacing and the characteristic dimensions of the sample [33].

A clear inverse relationship is observed: resistivity decreases significantly as RF power increases. Given that all films have a consistent thickness of approximately 800 nm, the variations in *ρ* are primarily driven by microstructural features (such as grain boundaries, defects, and film continuity), which exert a more dominant influence on charge transport in this thickness regime than in bulk materials.

At 50 W, the films exhibit the highest resistivity, increasing by several orders of magnitude at 25 mTorr. This behavior correlates directly with the structural analysis, which identified these films as predominantly amorphous or poorly crystallized. In this disordered state, limited atomic mobility during deposition leads to a high density of structural defects and grain boundaries that act as intense scattering centers. The resistive nature of these amorphous layers is likely governed by thermally activated hopping conduction through localized states [34], where the lack of long-range order disrupts efficient conductive pathways.

Conversely, films deposited at 60 W and 70 W show a marked reduction in resistivity. The improved crystallinity and larger grain size reduce the density of grain boundaries acting as electron-scattering centers, thereby facilitating more efficient charge transport. This behavior is consistent with the classical grain-boundary scattering model proposed by Mayadas and Shatzkes [34].

The influence of working pressure is also evident, particularly at lower power. At higher pressures, increased collisions in the plasma reduce the mean free path and the kinetic energy of the arriving species, leading to less dense films and a higher defect density. This effect is particularly evident at low power (50 W), where the combination of low energy and high-pressure results in the highest resistivity values. However, at 70 W, the increased energy input effectively compensates for these scattering effects, resulting in resistivity values that remain relatively stable over the pressure range studied.

While the lowest resistivity obtained (~10^−4^ Ω·cm) remains higher than the bulk value reported for Sb (~3.7 × 10^−5^ Ω·cm), this difference is expected for thin films and can be attributed to the combined effects of grain boundary scattering, surface/interface scattering, finite film thickness, and structural disorder. Although XPS revealed the presence of a thin native oxide layer, it is confined to the film surface and is therefore expected to contribute negligibly to the measured four-point probe resistivity.

Ultimately, these results establish a cohesive correlation between deposition parameters and the physical properties of the films, validating their potential as high-quality precursor layers for chalcogenide-based devices.

### 3.4. X-Ray Photoelectron Spectroscopy

The XPS results support the interpretation of the electrical behavior by confirming that the variations in resistivity are not related to changes in chemical composition. The analysis was performed at 60 W because this intermediate regime shows the strongest dependence on pressure in terms of microstructure and resistivity, as observed by XRD and electrical resistivity. While films deposited at 50 W exhibit high resistivity due to poor crystallinity and those at 70 W show more stable behavior due to enhanced grain growth, the 60 W condition is more sensitive to microstructural variations. The XPS spectra confirm a predominantly Sb composition across all pressures, indicating that changes in electrical properties are governed by structural factors, such as grain size and defects, rather than by chemical effects.

**Figure 6:**
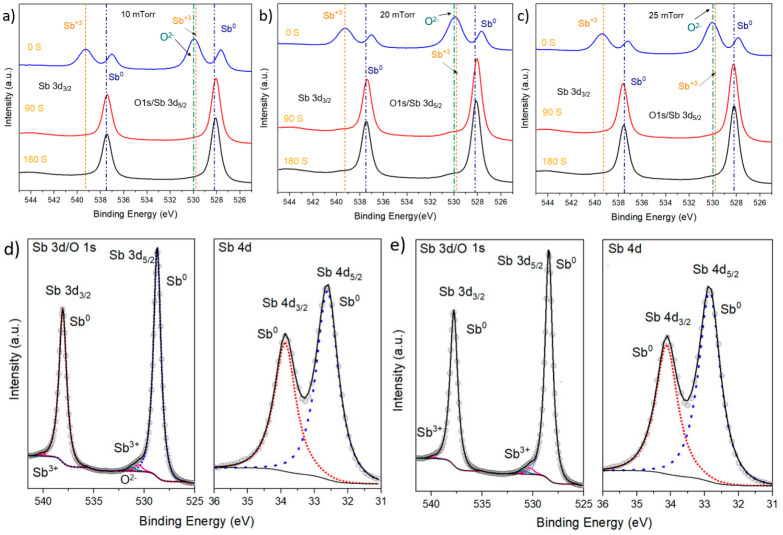
XPS spectra with different sputtering times of the sample deposited at 60 W at pressures of (**a**) 10 mTorr, (**b**) 20 mTorr, and (**c**) 25 mTorr, and the high-resolution and etch time spectra of Sb 3d_5/2_ and Sb 4d_5/2_ at pressures of (**d**) 10 mTorr and (**e**) 25 mTorr.

Figure 6a–e show the XPS spectra of Sb thin films deposited at 60 W under working pressures of 10, 20, and 25 mTorr. The Sb 3d/O 1s regions for different sputtering times (0, 90, and 180 s) are presented in (a–c), while (d,e) correspond to high-resolution spectra and peak deconvolution for representative samples. The analysis focuses on the Sb 3d, Sb 4d, and O 1s regions.

At the surface (0 s), the spectra exhibit a coexistence of antimony (Sb0) and oxidized species (Sb^3+^). While the primary peaks correspond to Sb, the presence of Sb^3+^ and O 1s signals indicates a thin oxide layer resulting from ambient exposure. To accurately resolve the chemical state despite the inherent overlap between the Sb 3d3/2 and O 1s peaks, the Sb 4d region was also analyzed. This high-resolution analysis confirms that the core of the films remains metallic, as evidenced by the characteristic Sb 3d doublet and its binding energy positions, which are consistent with reported reference values [35,36]. The depth profiling analysis reveals that even short sputtering times significantly reduce the intensity of the oxidized components, leaving the spectra dominated by Sb^0^. This behavior confirms that oxidation is confined to a surface layer of only a few nanometers, consistent with previous reports on Sb thin films [35]. To further evaluate the influence of RF power on the chemical state of the films, additional XPS depth-profiling measurements were performed on samples deposited at 50 and 70 W and at working pressures of 20 mTorr (See Supplementary Materials, Figures S2 and S3). Similar to the 60 W series, the spectra exhibit metallic Sb as the dominant chemical state beneath a thin native oxide layer. The oxide-related components are significantly suppressed after sputtering, confirming that oxidation is restricted to the surface. However, future angle-resolved XPS measurements could provide a quantitative determination of the native oxide thickness, further demonstrating that the oxide layer is confined to the film surface and does not significantly affect the electrical resistivity of the Sb thin films [37]. No substantial differences in chemical composition were detected between the 60 W, 50 W, and 70 W films, indicating that the pronounced changes in electrical resistivity observed at higher RF powers are predominantly associated with microstructural evolution rather than with changes in chemical state. In summary, the XPS results demonstrate that the films are predominantly metallic and chemically stable over the pressure range studied. This confirms that the variations in structural, morphological, and electrical properties are driven by microstructural evolution (such as grain size and texture) rather than changes in chemical stoichiometry, further validating these films as suitable precursor layers for chalcogenide devices.

## 4. Discussion

The microstructural and electrical trends observed in this work are consistent with reports on sputtered Sb_2_Se_3_ and Sb_2_S_3_ precursor layers, in which enhanced crystallinity and grain coalescence similarly reduce resistivity and improve charge transport pathways. The lowest resistivity achieved here (10^−4^ Ω·cm) approaches values reported [2,23] for optimized chalcogenide absorbers, underscoring the suitability of Sb thin films as a template for subsequent conversion processes. Importantly, the ability to tune grain orientation from (003) to (104) planes provides a pathway to engineer interfacial properties in Sb-based heterostructures, which is critical for optimizing carrier mobility in photovoltaic and thermoelectric devices. Alongside orientation control, substrate preparation has also been shown to play a decisive role: in situ plasma or thermal cleaning prior to deposition can modify the surface and interface quality, thereby influencing texture development and electrical performance [38]. Beyond these immediate implications, the systematic control of sputtering parameters demonstrated here establishes a reproducible framework for tailoring Sb thin films toward diverse optoelectronic applications. Future investigations should address substrate heating, interface engineering, and long-term stability under ambient exposure, as these factors will ultimately determine the scalability and industrial viability of Sb films in advanced device architectures. The pressure-driven transition from (003) to (012) preferred orientation observed at 60 W can be rationalized in terms of adatom thermalization: at higher working pressures, increased gas-phase collisions reduce the kinetic energy of sputtered Sb atoms, favoring the nucleation of lower-surface-energy planes. This mechanism is analogous to that reported for sputtered Sb_2_Se_3_ films, where pressure-controlled texture was shown to critically influence grain boundary character and, consequently, minority carrier lifetime. From a device integration perspective, films deposited at 70 W and 10 mTorr exhibit the most favorable combination of high crystallinity, (104)-oriented texture, and low electrical resistivity, making them promising precursor layers for the future fabrication of Sb_2_S_3_ and Sb_2_Se_3_ absorber materials. In contrast, the amorphous films obtained at 50 W may be advantageous for applications that require smooth and homogeneous precursor layers prior to thermal treatment, where a disordered structure could promote a more isotropic conversion. Although sulfurization and selenization were beyond the scope of the present work, evaluating how these distinct precursor microstructures influence the conversion process and the resulting device performance represents an important direction for future research.

## 5. Conclusions

This study establishes a comprehensive map of the structural and electrical evolution of RF-Sputtered Sb thin films, revealing that their functional properties are a direct consequence of a tunable microstructural regime. The transition from an amorphous, high-resistivity state to a polycrystalline, conductive rhombohedral phase is primarily governed by RF power, which serves as the primary parameter controlling growth kinetics and crystallinity.

A key finding is that these variations in electrical transport are structurally (rather than chemically) driven. As confirmed by XPS, the films maintain a stable metallic character, indicating that the observed reduction in resistivity is primarily the result of grain growth, improved connectivity, and reduced charge-carrier scattering. Working pressure serves as a secondary modulating factor, enabling fine-tuning of defect density and grain orientation by controlling plasma energy.

Beyond providing fundamental insights into growth mechanisms, this work identifies specific processing windows critical to the design of Sb-based precursor layers. The ability to precisely tailor the crystallinity and surface morphology of these films provides a strategic framework for developing high-efficiency chalcogenide-based optoelectronics. Future efforts focusing on substrate temperature and interface engineering will further expand the integration of these optimized layers into advanced device architectures.

## Figures and Tables

**Table 1:** Sputtering parameters for thin film deposition.

Deposition Conditions	Values
Base pressure	1 × 10^−5^ Torr
Substrate temperature	Room temperature
Pressure work	10, 20, and 25 mTorr
Sputtering power	50, 60, and 70 W
Ar flow rate	3.6 SCCM
Target-Substrate distance	4.5 cm

## Data Availability

The original contributions presented in this study are included in the article/Supplementary Material. Further inquiries can be directed to the corresponding authors.

## Supplementary Materials

The following supporting information can be downloaded at: https://www.mdpi.com/article/10.3390/ma19143119/s1. Figure S1: Preliminary XRD patterns of Sb thin films deposited at 10 mTorr and RF powers between 30 and 70 W, showing the amorphous-to-crystalline transition with increasing RF power. Figure S2: XPS depth profiles of Sb thin films deposited at a working pressure of 20 mTorr: (a) 50 W and (b) 70 W. The elemental distributions of Sb, O, and Si as a function of etching time show the presence of a surface oxide layer and a Sb-rich bulk region. Figure S3: High-resolution XPS spectra of Sb thin films deposited at 20 mTorr. (a) O 1s and (b) Sb 4d regions for the 50 W film; (c) O 1s and (d) Sb 4d regions for the 70 W film. The spectra reveal contributions associated with oxidized antimony species at the surface and metallic Sb in the bulk of the films. Table S1: Average resistivity values and corresponding standard deviations of the deposited films as a function of RF power and working pressure.
