# Supplementary material for: Influence of RF Sputtering Pressure and Power on the Microstructure of Sb Thin Films
Source: Materials (Basel). 2026 Jul 21;19(14):3119. doi: 10.3390/ma19143119 (PMC13413663; doi:10.3390/ma19143119)
Supplement: Supplementary file 1 [file materials-19-03119-s001.zip › materials-4440898-supplementary.pdf]

Supplementary Materials

Influence of RF Sputtering Pressure and Power on the Microstructure of Sb Thin Films

Sheyda Uc-Canche [1,2,*], Eduardo Camacho-Espinosa [3], Mariely Loeza-Poot [1], Ricardo Mis-Fernández [1] and Eduardo Flores [1,*]

1 Departamento de Física Aplicada, Centro de Investigación y de Estudios Avanzados CINVESTAV-Unidad Mérida, Mérida 97310, Mexico
2 División Industrial, Universidad Tecnológica Metropolitana, Mérida 97279, Mexico
3 Departamento de Energías Renovables, Universidad Abierta y a Distancia de México, México City 03330, Mexico
* Correspondence: sheyda.uc@cinvestav.mx (S.U.-C.); eduardo.flores@cinvestav.mx (E.F.)

This supplementary material contains additional structural and chemical characterization data supporting the results discussed in the main manuscript.

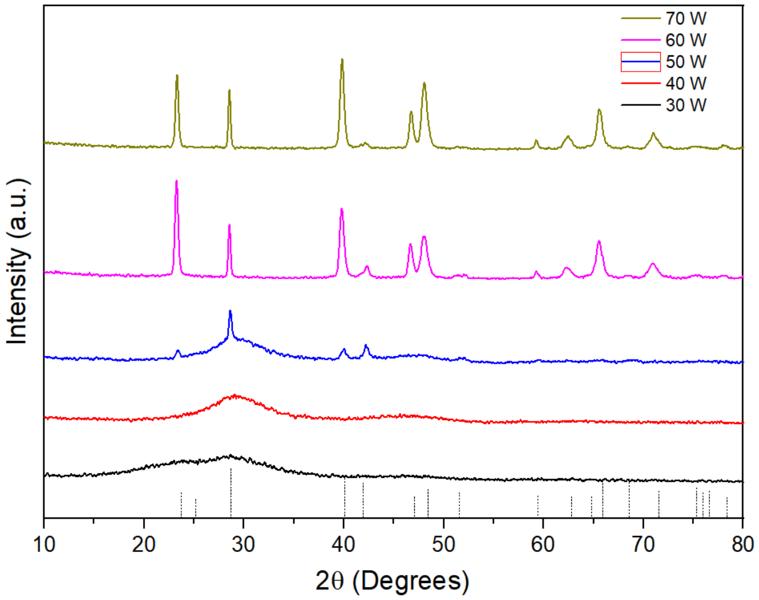

Figure S1. Preliminary XRD patterns of Sb thin films deposited at 10 mTorr and RF powers between 30 and 70 W, showing the amorphous-to-crystalline transition with increasing RF power.

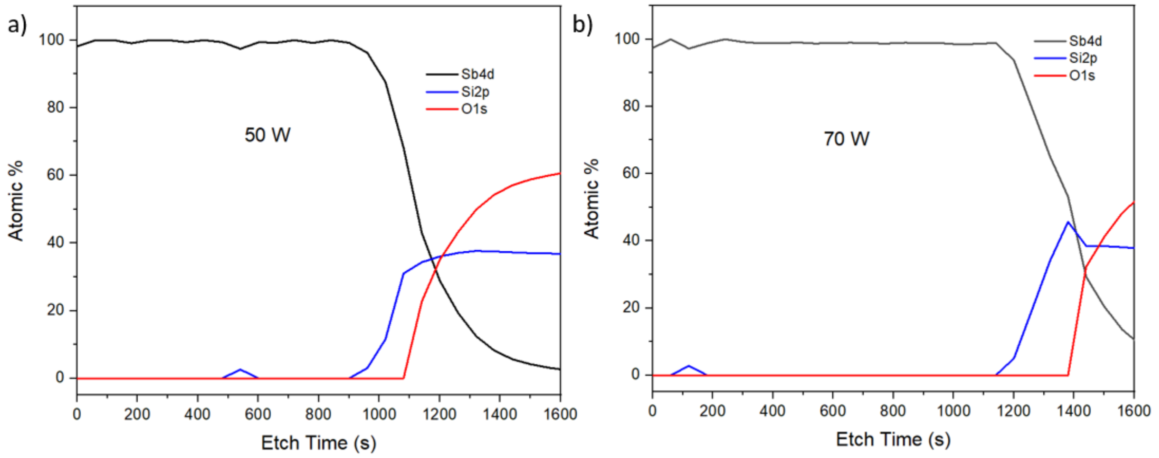

Figure S2. XPS depth profiles of Sb thin films deposited at a working pressure of 20 mTorr: (a) 50 W and (b) 70 W. The elemental distributions of Sb, O, and Si as a function of etching time show the presence of a surface oxide layer and a Sb-rich bulk region.

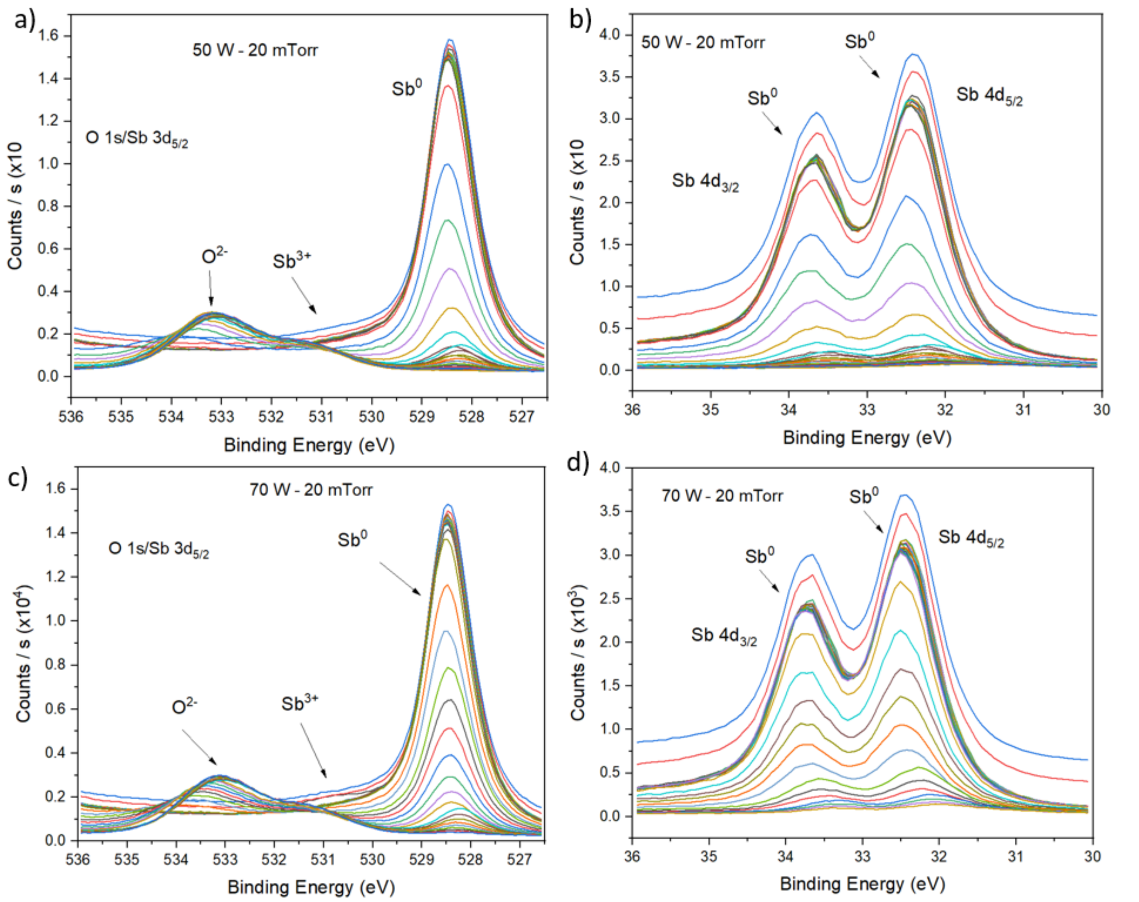

Figure S3. High-resolution XPS spectra of Sb thin films deposited at 20 mTorr. (a) O 1s and (b) Sb 4d regions for the 50 W film; (c) O 1s and (d) Sb 4d regions for the 70 W film. The spectra reveal contributions associated with oxidized antimony species at the surface and metallic Sb in the bulk of the films.

Table S1. Average resistivity values and corresponding standard deviations of the deposited films as a function of RF power and working pressure.

RF Power (W)	Pressure (mTorr)	Average resistivity (Ω·cm)	Standard deviation (Ω·cm)
50	10	1.10E-03	1.0E-04
	20	1.02E-03	1.0E-04
	25	1.06E-01	1.0E-02
60	10	4.49E-04	2.0E-05
	20	5.79E-04	2.0E-05
	25	4.76E-04	2.0E-05
70	10	2.32E-04	1.0E-05
	20	3.83E-04	2.0E-05
	25	2.72E-04	2.0E-05
